# The effects of exercise during pregnancy on the newborn’s brain: study protocol for a randomized controlled trial

**DOI:** 10.1186/1745-6215-13-68

**Published:** 2012-05-29

**Authors:** Elise L LeMoyne, Daniel Curnier, Samuel St-Jacques, Dave Ellemberg

**Affiliations:** 1 Department of Kinesiology, University of Montreal, 2100 Edouard Montpetit, Montreal, Canada; 2 Centre de Recherche En Neuropsychologie Et Cognition (CERNEC), Montreal, Canada

**Keywords:** Event-related potentials, Exercise, Mismatch negativity, Newborn, Pregnancy

## Abstract

**Background:**

It is generally accepted that an active lifestyle is beneficial for cognition in children, adults and the elderly. Recently, studies using the rat animal model found that the pups of mothers who exercised during pregnancy had increased hippocampal neurogenesis and better memory and learning abilities. The aim of this report is to present the experimental protocol of a study that is designed to verify if an active lifestyle during pregnancy in humans has an impact on the newborn's brain.

**Methods:**

60 pregnant women will be included in a randomized controlled study. The experimental group will be asked to exercise a minimum of 20 minutes three times per week, at a minimal intensity of 55% of their maximal aerobic capacity. The control group will not be exercising. The effect of exercise during pregnancy on the newborn's brain will be investigated 8 to 12 days postpartum by means of the mismatch negativity, a neurophysiological brain potential that is associated to auditory sensory memory. We hypothesize that children born to mothers who exercised during their pregnancy will present shorter latencies and larger mismatch negativity amplitudes, indicating more efficient auditory memory processes.

**Discussion:**

As of September 2011, 17 women have joined the study. Preliminary results show that the experimental group are active 3.1 ± 0.9 days per week while the control group only exercise 0.8 ± 0.6 days per week. The results of this study will present insight on fetal neuroplasticity and will be a valuable tool for health professionals who wish to encourage pregnant women to exercise.

**Trial registration:**

ClinicalTrials.gov registration: NTC01220778

## Background

Accumulating data supports that an active lifestyle is beneficial for cognitive functioning in children [1-3], adults [4] and the elderly [5]. For example, Davis *et al.*[6] found that 7- to 11-year-old children who exercised 40 minutes per week for 15 weeks performed significantly better on a test of executive functions than children who exercised for 20 minutes or less.

A recent group of studies using various exercise protocols with the rat animal model also suggests that maternal exercise during pregnancy has a beneficial influence on the development of the fetal brain that ultimately leads to functional changes for the newborn rat pups [7-14]. Parnpiansil *et al.* report that the pups of mothers who exercised during pregnancy had increased hippocampal neurogenesis as well as better memory and learning abilities. Specifically, 30 minutes of treadmill running five days per week during gestation was associated with better spatial learning in the multiple t-maze [8]. Further, Akhavan *et al.* found that both voluntary running and forced swimming during gestation increased the quantity of cells in the cornus ammonis 1 (CA1) and dentate gyrus regions of the hippocampus of the rat pups and facilitated learning in the Morris Water Maze [12].

Few studies with humans have investigated the relationship between physical activity during pregnancy and the offspring’s cognitive functioning. In a series of seminal studies, Clapp and his team compared the children of women who continued to be active during their pregnancy to those of closely matched women who voluntarily stopped exercising during their pregnancy. All participants were recruited before their pregnancy and their exercise performance, dietary intake, weight gain and physiologic responses were monitored throughout their pregnancy. The active women ran, performed aerobics or cross-country skied three or more times a week for 20 minutes or more at an intensity above 55% of their maximal aerobic capacity (VO_2_max). Five days after their birth, the offspring of the mothers who were active during their pregnancy scored higher on the orientation and state regulation subscales of the Brazelton Neonatal Behavioral Assessment Scales. This reflects the ability to orient to stimuli from the environment and the ability to self-regulate after the presentation of sound and light stimuli [15]. Further, in a separate study, at the age of one year, the children of the active group had a significantly higher score on the psychomotor scale of the Bayley Scales of Infant Development [16] and, in yet another study, at the age of five years old they had better results on tests of general intelligence and oral language [17]. Important as they may be, these studies do have some notable limitations that need to be considered in the interpretation of their results. First, no direct measure of brain or neuronal change is provided. The data from the neonates suggest that exercise during pregnancy influences certain of the newborns’ behaviors, but unlike the data from the animal studies they do not provide direct evidence of changes in brain activation. Second, the results of the two older age groups could have been influenced by differences in the upbringing provided by the mothers from each group. As acknowledged by Clapp and his team, ‘perhaps there is something fundamentally different about the maternal-child interaction in the families of the women who chose to maintain their exercise throughout pregnancy’ [17]. The goal of the present study was to extend the work of Clapp and his colleagues by comparing the neurophysiological brain potentials of babies born to women who were active during their pregnancy to those of babies born to women who were sedentary.

Recent studies in humans have been able to directly investigate functional neural activation in newborns by means of the mismatch negativity (MMN), a component of the auditory event-related potential [18,19]. The MMN wave is elicited by presenting a rare auditory stimulus in a series of repetitive frequent standard sounds. The MMN has been associated with auditory sensory memory and several authors believe that it is an objective measure of the newborn’s cognitive status [20]. For example, earlier and stronger MMN waveforms indicate greater developmental maturity [21], whilst a longer latency and a smaller amplitude are associated with slowed development in children with cleft palate [22] or autism [23].

To further our understanding of the effects of exercise during pregnancy on the development of the human brain, we propose to use a randomized intervention protocol and a direct measure of neonatal brain functioning. Pregnant women will be randomly allocated to an active group or an inactive control group and the latency and amplitude of the MMN evoked-potential will be recorded in their newborns. Given that the main finding from animal studies is an improvement in learning and memory in the offspring of active mothers, we hypothesize that children born to mothers who exercised during their pregnancy will present shorter latencies and larger MMN amplitudes compared with babies born from non-exercising mothers.

## Methods

### Design and ethical considerations

This will be a single-center randomized controlled trial with a two-group design: one group exercising regularly and a non-exercising control group.

All study procedures have been approved by the Health Sciences Research Ethics Committee of the University of Montreal, Montreal, Quebec, Canada. Informed consent will be obtained after participants receive verbal and written information about the purpose and procedures involved in the study and before any data collection begins.

### Recruitment and participants

All obstetricians and gynecologists of the Montreal area were contacted with the help of the Collège des Médecins du Québec and were asked to distribute brochures to women who meet the inclusion criteria. Advertisements were also placed on Facebook and articles were published in the university paper and on several specialized blogs. Recruitment will continue until 60 pregnant women have joined the study, 30 in the exercise group and 30 in the control group.

Inclusion and exclusion criteria were chosen to minimize individual differences among participants and to ensure the women’s safety (see Table1). Participants will be withdrawn from the study if exclusion criteria are met during the course of the study or if they withdraw their consent.

**Table 1:** Inclusion and exclusion criteria for the Healthy Mom, Bright Child study

**Inclusion criteria**	**Exclusion criteria**
First trimester of pregnancy	Use of alcohol, cigarettes or illegal drugs
20 to 35 years of age	Health complications
Pre-pregnancy body mass index between 18 and 25	Unable or unwilling to breastfeed
No known health conditions	Excessive or insufficient weight gain (25 to 35 lbs according to pregnancy weight gain recommendations) [24]
Use of folic acid	Non-compliance with the experimental protocol

### Procedure and timeline

**Figure 1:**
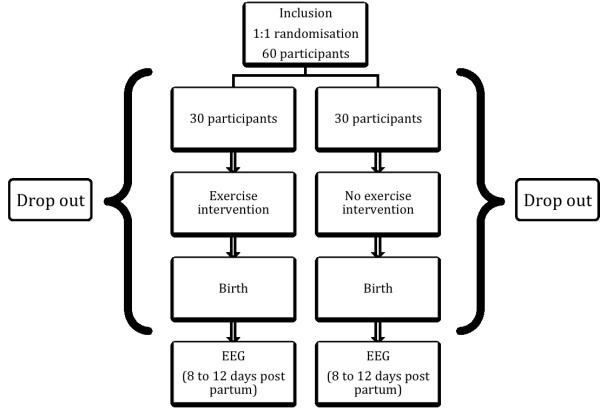
Study design.

Women will join the study prior to their 13^th^ week of pregnancy to receive the intervention during the entire second and third trimesters. Gestational age will be determined from the menstrual history. During the first meeting, women will be informed that they will be randomly assigned to the active or control group. Study design is presented in Figure1. They will be clearly informed of the characteristics of each group and of what is expected of them. To avoid any auto-selection bias, they will be assigned to a group only after they have provided their informed consent.

Two interventions will be offered: individual recommendations on health habits during pregnancy; and individual recommendations on exercising during pregnancy. To ensure that each group has similar knowledge about health habits during pregnancy and that there is the same amount of one-on-one contact with all participants, both groups will receive the first intervention. Only the active group will receive the second intervention, recommending at least three sessions of exercise per week (see section on Exercise intervention). Participants in the control group will not be given any exercise counseling and will be informed they will be excluded from the study if they exercise more than once per week.

**Figure 2:**
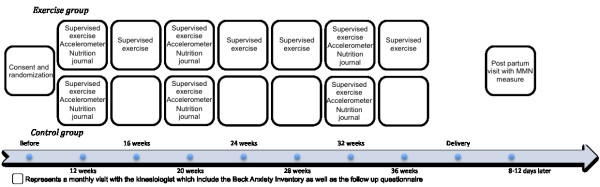
**Timeline of the experimental protocol.** Monthly visits with the kinesiologist include the Beck Anxiety Inventory as well as health and habit questions.

Starting at the beginning of the second trimester, all women will complete a daily log and wear a pedometer for the entire duration of the study. Women from both groups will meet monthly with the same kinesiologist and will receive standardized advice and feedback based on their daily log. During that meeting, all the women will complete a mood questionnaire and the women who are part of the active group will complete an exercise session in order to learn how to characterize their at-home exercise for the daily log. The women who are part of the control group will complete the same exercise session but only once per trimester. During each trimester women from both groups will fill out a three-day nutrition journal and during the second and third trimesters they will wear an accelerometer and a Polar heart rate monitor for seven days and three nights. The timeline of the experimental protocol is presented in Figure2.

### Interventions

#### Health habits intervention

Women in both groups will be given the *Sensible Guide to a Healthy Pregnancy*[25] created by the Public Health Agency of Canada*.* The guide contains information on the following health habits during pregnancy: nutrition, folic acid, alcohol consumption, physical activity, smoking, oral health and emotional health.

### Exercise intervention

Women randomly assigned to the exercise group will be asked to exercise a minimum of three times per week for 20 minutes each time at a minimal intensity of 55% of their VO_2_max, as per the protocol of Clapp and colleagues [15]. Intensity will be measured using the 6 to 20 Borg perceived exertion scale [26], given that it is accepted that 12 on the Borg scale corresponds to 55% of an individual’s VO_2_max [27].

### Measurements

#### Exercise intensity

Participants in the exercise group will perform a supervised exercise session on a cycle ergometer every month (Corival, Lode B.V., Groningen, The Netherlands) to learn the use of Borg’s perceived exertion scale. To allow the participants to easily identify the minimal intensity required for their at-home exercise, target intensity will correspond to 12 on the Borg scale (that is, 55% of their VO_2_max). The entire session will last 30 minutes, including a 5 minute warm up and a 5 minute cool down. Participants in the control group will be asked to complete a similar supervised session once per trimester to allow them to measure intensity in the event that they do exercise.

### Primary outcome measures

The MMN will be measured as early as 8 to 12 days after birth in order to isolate the effects of exercise during pregnancy from the possible effect of postpartum health habits. Brain activity will be recorded using the 124 electrodes Geodesic system of EGI Acquisition Systems (Eugene, Oregon, USA). Because this is safe, easy to install and provides good spatial resolution, it is commonly used in studies with newborns [28]. Electrode impedance will be kept below 50 kΩ, which is the recommended level for high input impedance amplifiers [29]. Each electrode will be referenced to Cz and an electrode placed anterior to Pz will serve as the ground. The electroencephalography (EEG) signal will be amplified with Net Amps 200 amplifier (EGI, Eugene, OR, USA) and a band-pass filter will be set at 0.1 to 100 Hz. The signal will be digitalized at 250 Hz and the data will be recorded with Net Station software (EGI). Recordings will take place in a dimly light electromagnetic-isolated and sound-attenuated room.

EEG recordings will be analyzed using Brain Vision Analyzer software, version 1.05 (Brain Products, Munich, Germany). The data will be filtered off-line with a digital band-pass filter of 0.1 to 15 Hz and 24 dB/octave. Blinks and ocular movements will be corrected with independent component analysis. The data will be re-referenced to average mastoids (A1-A2). The MMN will be determined using a deviant-minus-standard subtraction and by identifying the first peak within the 100 to 350 ms time window of the difference wave. Mean MMN amplitudes will be calculated as a mean voltage of 40 ms intervals, centered at peak latency over the midline and laterally for the fontal, central and parietal regions (that is, F3, Fz, F4, C5, C3, C1, Cz, C2, C4, C6, P5, P3, P1, Pz, P2, P4, P6).

We will use the protocol developed by Ceponiene *et al.*, which has been shown to successfully measure the MMN in newborns [30]. Three blocks of 500 sounds will be presented. The stimuli will be harmonic tones as they have to elicit a robust and reliable signal in adults and children [30]. The standard stimulus, presented in 85% of the trials, will be composed of the fundamental frequency of 500 Hz and its first two harmonics (500, 1,000 and 1,500 Hz). The intensity of the second and third components will be reduced by 3 and 6 dB, respectively. The deviant stimulus, presented in 15% of the trials, will consist of the fundamental frequency of 750 Hz and its first two harmonics (750, 1,500 and 2,250 Hz) with the same reductions of 3 and 6 dB, respectively, for the last two components. All sounds will be presented at 70 dB sound pressure level for a period of 100 ms, with an 800 ms stimulus onset asynchrony.

### General information questionnaire

During the first visit, all participants will be asked to answer questions regarding their and their partner’s professional, economic, educational and health status as well as a family history of hereditary disorders. This questionnaire also documents the details of the participants’ pregnancy.

Monthly visits will include a follow-up questionnaire that will inquire about their health and general habits (for example, alcohol consumption, massages, acupuncture) to ensure they remain eligible to participate in the study as well as to document potential confounders between the two groups.

### Physical activity survey

Exercise habits for the previous year will be assessed using the Kaiser Physical Activity Survey (KPAS) and the first trimester’s exercise habits will be documented with a modified version of the KPAS that was validated for pregnancy [24].

### Daily log

Women will be asked to complete, on a daily basis, an online questionnaire containing 12 questions to document their exercise practice (type, duration, intensity), the quality of their sleep, and their use of medication and supplements.

### Pedometer

Women from both groups will wear a pedometer (Yamax Digi walker SW-200, Tokyo, Japan) on a daily basis for the entire duration of the study. This measure will verify that the exercise sessions performed by the women in the exercise group do not lead to a compensatory augmentation of sedentary behaviors, as has been documented in other exercise intervention studies [31]. Further, pedometer readings will provide an additional measure of physical activity with which to compare both groups.

### Mood questionnaires

Animal studies on the effect of exercise during pregnancy on the newborn’s brain suggest that a high level of maternal anxiety diminishes this effect [12]. Therefore, during their monthly visit, the women will be asked to fill out the Beck Anxiety Inventory [32].

### Accelerometer

All women will wear an ActiTrainer accelerometer (MTI-ActiGraph, Fort Walton Beach, FL, USA) for seven days and three nights during each trimester. This will allow us to verify the accuracy of their daily logs.

### Nutrition journal

Participants will also be asked to fill out a three-day nutrition journal for each trimester. They will be asked to document all of their food and liquid intake with details about the type and quantity of each food item.

### Medical records

A copy of the complete medical records will be obtained from each woman’s obstetrician at the postnatal visit to verify weight gain and standard pregnancy blood test results as well as details of the delivery.

### Preliminary results

As of September 2011, 17 women have given their consent and been randomly assigned to one of the two groups: nine in the exercise group and eight in the control group. Two women from the exercise group and three from the control group were excluded: two for having a miscarriage, one for being pregnant with twins, one for not complying with the experimental protocol, and one for withdrawing consent. Table2 presents the characteristics of all participants.

**Table 2:** Participant characteristics

	**Active group**	**Control group**
	**(n = 7)**	**(n = 5)**
Age (years)		
Mother	29 ± 3	28 ± 4
Father	31 ± 4	31 ± 6
Pre-pregnancy body mass index	24.9 ± 3.7	27.9 ± 5.8
Pre-pregnancy KPAS index	11.62 ± 1.39	10.93 ± 0.65
First trimester KPAS index	9.71 ± 1.19	8.97 ± 1.39
Mother	3 (3)	5 (5)
Father	5 (7)	3 (3)
Revenue ($)	80,000 to 100,000	60,000 to 80,000
(median class)	(40,000)	(40,000)

Data presented as the mean ± standard deviation, except for Education and Revenue, which are presented as the interquartile range. KPAS: Kaiser Physical Activity Survey.

Preliminary results indicate that all women completed the online daily log from week 13 until birth. The active women (n = 7) had journal entries for 23 to 28 weeks and exercised an average of 3.1 ± 0.9 days per week. Sessions lasted from 20 to 90 minutes, at an intensity ranging from 12 to 15 on the Borg scale. Activity type included aerobics, swimming, tennis, elliptical training, hiking, circuit training, Wii sports and rollerblading, with the most common being walking, cycling and water aerobics. Women in the control group (n = 5) had journal entries for 24 to 27 weeks and exercised an average of 0.8 ± 0.6 days per week. Sessions lasted from 20 to 40 minutes, at an intensity ranging from 12 to 13 on the Borg scale. Activities included cycling, water aerobics and swimming, walking being by far the most common.

### Analyses

#### Sample size calculations

To determine the number of participants that need to be included in the present study we completed power analysis from published data obtained by measuring MMN in newborns. MMN studies in newborns report changes in amplitude of up to 260% [30]. However, because no previous study observed the effect we are looking for, we chose to be conservative in our calculations and used an expected difference of 50%. We also used a standard deviation of 56% estimated from the data of frontal electrodes presented in the study by Ceponiene *et al.*, whose MMN protocol we will be using [30]. Thus, to reach statistical significance, 21 women are needed for each group (type I error rate = 0.05 and type II error rate 0.20 = 80% power). Assuming a 30% attrition rate, we want to enroll 30 participants for each group.

#### Statistical analyses

A series of analyses will be completed to determine parity between the groups. Specifically, t-tests will compare sociodemographic status, pre-pregnancy health status, pre-pregnancy and first trimester KPAS scores, nutrition journals, and scores on the anxiety scale between the control and the active groups. We will also complete a qualitative analysis of the medical records including standard blood test results and the details of delivery. A second series of t-tests will verify compliance with the experimental protocol by comparing the data from the daily exercise logs, the pedometers and the accelerometers of both groups.

Separate repeated measure analysis of variance (ANOVA) will be performed on the amplitude and latency data of the recorded brain waves. For each ANOVA, the between-subject factor will have two levels, corresponding to the exercise and control groups and the within-subject factors will be region (frontal, central and parietal) and laterality (left, central and right). Statistical analyses will be carried out using the SPSS software (SPSS 17.0, Chicago, IL, USA). A *P* <0.05 will be considered statistically significant.

## Discussion

To the best of our knowledge, this is the first randomized controlled trial examining the effects of exercise during pregnancy on the neuroelectric responses of the newborn’s brain. This will provide a direct measure of the influence of physical exercise during pregnancy on the developing brain. The results will contribute to our understanding of the effects of physical exercise during pregnancy on the newborn’s brain and provide some insight on fetal neuroplasticity (that is, how the brain of the fetus is influenced by its environment). Further, if the results of the study confirm our hypothesis, they should influence the way in which health professionals present and discuss exercise with pregnant women. Health professionals will not only encourage pregnant women to exercise for their own health, but also for the benefit of their child’s development.

The findings from the present study will also provide the empirical data to support future research that should concentrate on establishing the ideal volume of exercise needed to achieve the best effects for the newborn and that should aim to identify the biological mechanisms that are responsible for this effect.

### Methodological strengths

There are several strengths to the proposed protocol. First, we use validated and sensitive neuroelectric measures of the newborn’s brain activity. Also, the prospective randomized design will ensure that the participants in the active and the control groups are comparable for all other characteristics. We will verify group parity by comparing sociodemographic status, pre-pregnancy health status, pre-pregnancy and first trimester KPAS scores, nutrition journals, and scores on the anxiety scale. Further, the same kinesiologist will meet with all of the participants to ensure the standardization of the experimental protocol, including advice and feedback.

### Methodological weaknesses

Our protocol also has some limitations that will be taken into account in the interpretation of the data, but that are unlikely to influence the overall pattern of results. Our close monitoring of the participants’ daily exercise via the daily log and wearing of a pedometer will allow us to determine the amount of exercise completed by the experimental group and will allow us to eliminate members of the control group who do not conform to the non-exercising criteria.

We are aware that the MMN is not an intuitive measure for parents about the general functioning of their newborn. However, there are several reasons why we opted for this measure. First, although it may seem abstract, it is associated with specific behavioral processes including sensory memory and cognitive status [18-20]. Second, it is an objective measure of brain functioning in the newborn that is not subject to examiner bias or the child’s state of wakefulness [19]. There are no known behavioral equivalents to this measure that are as sensitive to the newborn’s brain functioning.

## Trial status

Participant recruitment for this trial is ongoing.

## Competing interests

The authors declare that they have no competing interests.

## Authors’ contributions

ELL conceived and designed the study, recruited and tested participants, carried out all interventions and drafted the manuscript. DC contributed to the design of the study and revised the manuscript. SSJ contributed to the design of the study. DE conceived and designed the study and revised the manuscript. All authors read and approved the final manuscript.
